# Application of a multilayer perceptron artificial neural network for identification of peach cultivars based on physical characteristics

**DOI:** 10.7717/peerj.11529

**Published:** 2021-06-17

**Authors:** Adel M. Al-Saif, Mahmoud Abdel-Sattar, Abdulwahed M. Aboukarima, Dalia H. Eshra

**Affiliations:** 1 College of Food and Agriculture Sciences, Department of Plant Production, King Saud University, Riyadh, Saudi Arabia; 2 Faculty of Agriculture, Pomology Department, Alexandria University, Alexandria, Egypt; 3 College of Food and Agriculture Sciences, Department of Agricultural Engineering, King Saud University, Riyadh, Saudi Arabia; 4 Agricultural Research Center, Agricultural Engineering Research Institute, Giza, Dokki, Egypt; 5 Faculty of Agriculture, Food Science and Technology Department, Alexandria University, Alexandria, Egypt

**Keywords:** Artificial neural network, Classification, Color, Grading, Identification, Peach, Physical properties, Sorting, Data mining, Sensitivity analysis

## Abstract

In the fresh fruit industry, identification of fruit cultivars and fruit quality is of vital importance. In the current study, nine peach cultivars (Dixon, Early Grande, Flordaprince, Flordastar, Flordaglo, Florda 834, TropicSnow, Desertred, and Swelling) were evaluated for differences in skin color, firmness, and size. Additionally, a multilayer perceptron (MLP) artificial neural network was applied for identification of the cultivars according to these attributes. The MLP was trained with an input layer including six input nodes, a single hidden layer with six hidden nodes, and an output layer with nine output nodes. A hyperbolic tangent activation function was used in the hidden layer and the cross entropy error was given because the softmax activation function was functional to the output layer. Results showed that the cross entropy error was 0.165. The peach identification process was significantly affected by the following variables in order of contribution (normalized importance): polar diameter (100%), *L*^∗^ (89.0), *b*^∗^ (88.0%), *a*^∗^ (78.5%), firmness (71.3%), and cross diameter (37.5.3%). The MLP was found to be a viable method of peach cultivar identification and classification because few identifying attributes were required and an overall classification accuracy of 100% was achieved in the testing phase. Measurements and quantitative discrimination of peach properties are provided in this research; these data may help enhance the processing efficiency and quality of processed peaches.

## Introduction

The peach (*Prunus persica* L.), a member of the Rosaceae family, is one of the main fruit crops in the temperate regions of the world (Ma et al., 2014; Rawandoozi et al., 2020). Peach fruit is rich in nutrients, particularly vitamins A, B, and C, carbohydrates, and some minerals (*Khalifa & Hamdy, 2018*). The quality of fresh peaches is determined by parameters such as fruit size, an important quality attribute (*García, Casierra-Posada & Contreras, 2018*) that is considered a requisite of quality by consumers (*Morais et al., 2017*), and color (*Matias et al., 2016; Matias et al., 2017; Silva et al., 2016*). For the latter, Matias et al. (2016) reported strong correlations among carotenoid content and hue angle for pulp color and fruit mass, suture diameter, equatorial diameter, and polar diameter, and in 28 peach cultivars. In addition, peach ripening is related to physical changes, particularly color changes, which are known to be associated with nutritional value (*Kader, 2008*).

For marketing purposes, fruits are usually classified based on their external quality parameters. Thus, visual inspection is most frequently used for classification because of its practicability and simplicity (Al Ohali, 2011). Accurate identification of fruit varieties is also an important stage in fruit production. To differentiate one fruit variety from another, basic attributes such as texture, color, and shape are used (*Rajasekar & Sharmila, 2019*). Thus, visual perception is the key to choosing healthy and nutritious foods. As mentioned above, color is often used as an indicator of food quality, defects, and grade (*Comert, Mogol & Gokmen, 2020*). The CIELAB color space, consisting of *L*^∗^, *a*^∗^, and *b*^∗^ coordinates, is normally used to define the color of foods. *L*^∗^ is a measure of lightness on a scale of zero to 100 (with zero and 100 representing black and white, respectively). The *a*^∗^ axis ranges from red (positive values) to green (negative values), whereas the *b*^∗^ axis ranges from yellow (positive values) to blue (negative values) (*Aälvarez-Fernaändez et al., 2003*). In the field, fruit color can be affected by the extent of light penetration through the tree canopy (*Fallahi et al., 2009*) and fruit quality characteristics are also affected by the position of the fruit within the canopy (*Lewallen, 2000; Bonora, Stefanelli & Costa, 2013*).

Fruit identification can be considered a procedure of allocating an object to a group or class according to a high level of evidence using an appropriate classification algorithm. Such classification algorithms can use statistical learning, machine learning, or other methods (*Cornejo & Pedrini, 2016; Wang et al., 2016*). Research into the classification of fruits has previously been presented in numerous studies (*Rocha et al., 2010; Al Ohali, 2011; Rajasekar & Sharmila, 2019*); however, classification remains a challenging task for researchers. Shahin et al. (2002) pointed out that quality assessment of apples with computer vision techniques could be achieved through development a system composed of line scan x-ray imaging and artificial neural network (ANN) classifier. The system was able to detect the fresh fruit business. The results revealed that when the ANN classifier was used to sort apples based on old bruises, it achieved an accuracy of 90% for Red delicious apples and 83% for golden delicious apples. For new bruises, the accuracy was approximately 60% for both Red delicious and golden delicious apples.

Machine learning technology has the potential to be applied for the classification of agricultural products. For example, machine learning classification techniques can be used to identify fruits based on three basic features: color, shape, and texture (*Rajasekar & Sharmila, 2019*). In bananas, *Juncai et al. (2015)* employed a support vector machine to classify ripening stages, with the color values *L*^∗^, *a*^∗^, and *b*^∗^ used as input data; classification accuracy using the support vector machine based on the radial basis function kernel reached 96.5%, which was higher than that for the linear function kernel. For peach classification specifically, *Esehaghbeygi et al. (2010)* presented a peach fruit sorting system in which machine vision classified peaches into three quality classes: red–yellow, yellow–red, and yellow. These authors also employed size as a peach classification feature, with the accuracy of classification reaching 90% and 96% according to color and size features, respectively. In addition, Alipasandi, Ghaffari & Alibeyglu (2013) employed image processing and ANN classification to identify three peach cultivars, namely Shalil Nectarine, Anjiri, and Elberta cultivars. These authors extracted 12 color components and three shape attributes using a machine vision system and applied these as inputs to classify peaches into three categories with classification accuracy rates of 98.5% and 99.3% for mature and immature fruits, respectively. In another study, Bermúdez, Padilla & Torres (2013) introduced a machine vision system for classification of the Iranian saffron peach. Peach size and color were measured to categorize peaches into three quality classes of red-yellow, yellow-red, and yellow. Experimental results showed that using color as classification features achieved accuracy around 90%. Moreover, *Bhatt, Pant & Singh (2014)* developed ANN-based apple classifier. The complete system was composed of two parts. In the first part, input (surface level apple quality parameter) from the different sources was collected by the software developed in Visual Basic through different input device like web camera, weight machine, etc. In the second part, the input data were used by ANN simulator to classify the apple according to their quality. A low level of error prediction confirmed the fact that the ANN model was an effective model of the apple quality estimation. Also, there was not any misclassification during testing.

The applying of machine learning technologies in agriculture for plant identification and crop type prediction, particularly those based on ANN models can provide essential information for decision support in agriculture (*Zhang et al., 2019*). However, Guo et al. (2016) used a support vector machine and classification attributes extracted using near-infrared diffuse reflectance spectroscopy to establish a peach cultivar identification model; the accuracy of their support vector machine reached 100%. To identify three species of peach, *El-kahlout & Abu-Naser (2019)* used a deep-learning convolutional neural network model with a dataset containing 2,306 images (70% and 30% of these images were used for training and validation, respectively); the trained model reached an accuracy of 100% with the testing dataset. *Koklu & Ozkan (2020)* developed a computer vision system to distinguish seven different registered varieties of dry beans with similar features in order to obtain uniform seed classification. Bean images obtained by computer vision system were subjected to segmentation and feature extraction stages, and a total of 16 features; 12 dimensions and four shape forms, were obtained from the images. Multilayer perceptron, support vector machine, k-nearest neighbors, decision tree classification models were created with 10-fold cross validation. Overall correct classification rates have been determined as 91.73%, 93.13%, 87.92% and 92.52% for multilayer perceptron, support vector machine, k-nearest neighbors and decision tree, respectively.

In peach production, peach cultivars should ideally be harvested when the fruit reaches maturity. At this stage, the peaches are appropriate for the fresh fruit market. In the present study, we used nine peach varieties that had reached maturity and carefully examined differences in peach skin color, firmness, and polar and cross fruit lengths. These parameters were considered as external quality indicators to create a classifier model that provided information for peach cultivar identification purposes. The results of this research could improve recognition of subclasses for peach marketing. In addition, we investigated the possible use of an ANN classifier based on a combination of skin color, size, and firmness attributes for the identification of peach cultivars.

## Materials & Methods

### Experimental site and plant materials

**Figure 1:**
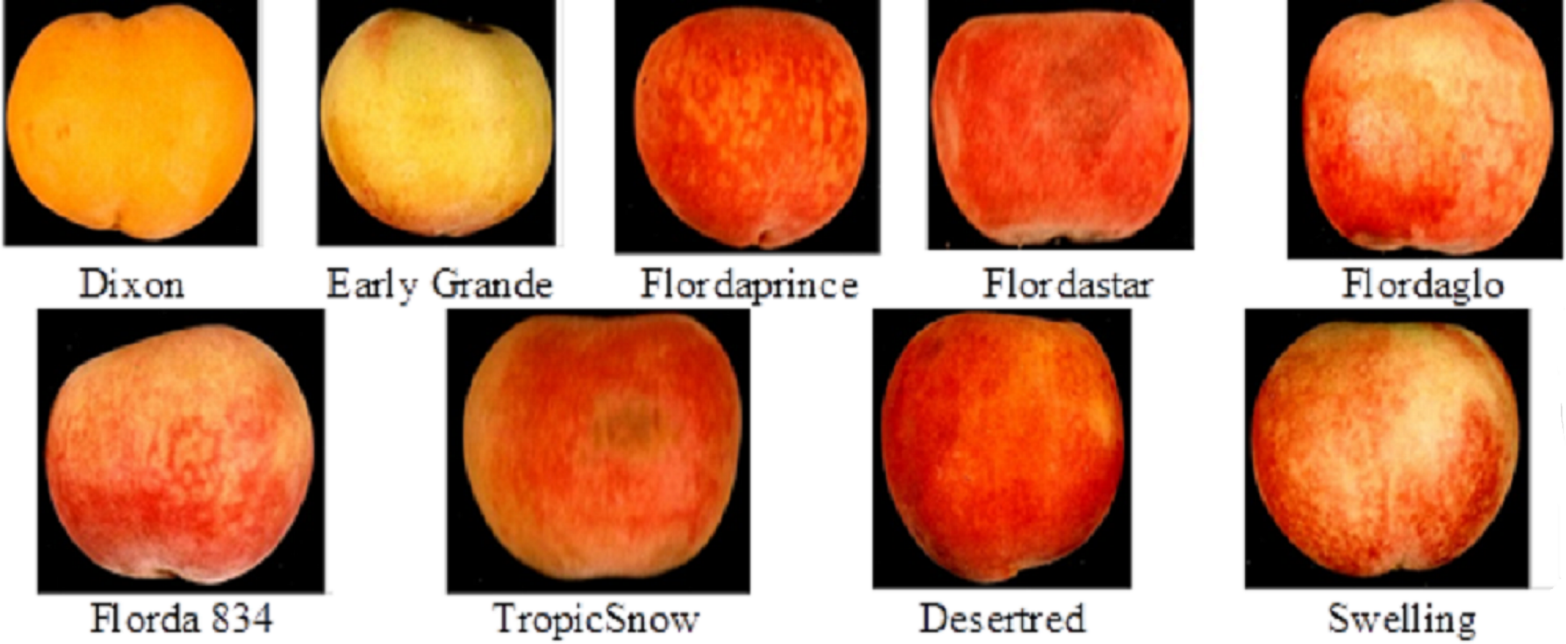
The nine peach (*Prunus persica L.*) cultivars investigated in this study.

This research was conducted during 2019 using nine peach cultivars: Dixon, Early Grande, Flordaprince, Flordastar, Flordaglo, Florda 834, TropicSnow, Desertred, and Swelling (Fig. 1).The peach trees, which were eight years old, were grown in sandy soil (pH of 7.7–7.8) in a private commercial orchard near the Pico Company, El-Behera Governorate, Egypt. The peach trees were budded in Nemaguard rootstock spaced at 3.5 × 5 m apart. All tree cultivars were similarly pruned, trained, and thinned; thus, color descriptions accurately reflect the relative colors of the investigated peach fruits. The orchard was irrigated by drip irrigation system. Technical and horticulture processes such as fertilization, disease and pest control were carried out regularly based on standard (El-Etreby, 1996; Ikinci & Bolat, 2018). Four trees (as uniform as possible) were chosen from each peach cultivar to collect fruit samples.

Fruits were harvested when fully mature according to commercial practice and immediately transported to the laboratory located at the Department of Food Science and Technology, Faculty of Agriculture, Alexandria University, Alexandria, Egypt. All fruits were harvested at the ripe stage and samples with defects (sunburn, cracks, bruises, and/or cuts in the husk) were discarded.

### Measurements of fresh peach fruit properties

Peaches were neither washed nor brushed before being measured. For each cultivar, 20 random fruits were used to define the flesh firmness of each of the two sides using an Effegi pressure tester with a 5/16-inch plunger (Effegi, 48011 Alfonsine, Italy); flesh firmness was expressed as Lb/inch^2^. Fruit diameters, polar and cross, were measured in mm immediately after harvesting using a digital caliper. According to Commission Regulation (EC) no. 1861/2004, 56 mm is the minimum diameter for a fruit to be considered in the “extra” category, which can be subdivided into different categories as follows: AAAA (diameter > 90 mm), AAA (90 mm > diameter ≥ 80 mm), AA (80 mm > diameter ≥ 73 mm), A (73 mm > diameter ≥ 67 mm), B (67 mm > diameter ≥ 61 mm), C (61 mm > diameter ≥ 56 mm), and D (i.e., not in the “extra” category; 56 mm > diameter ≥ 51 mm) (*Mirás-Avalos et al., 2017*).

**Figure 2:**
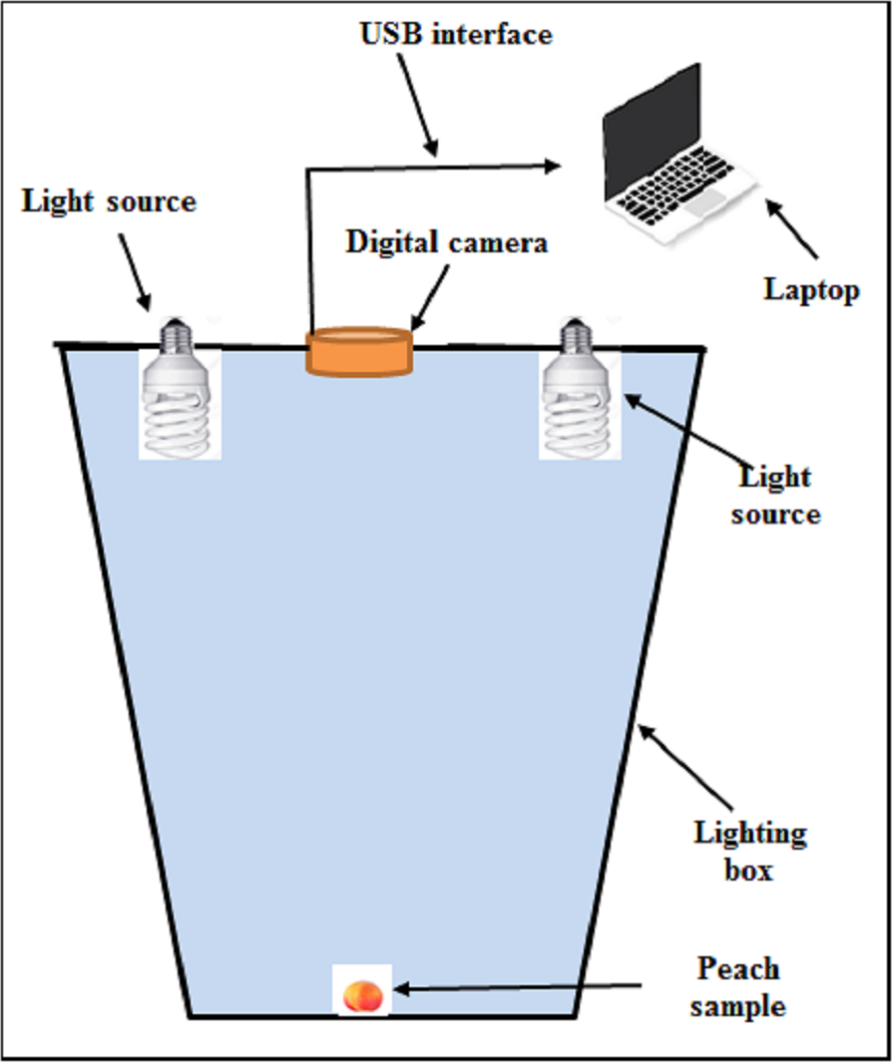
Lighting box apparatus used for the capture of peach images.

As the measurement of skin color is nondestructive, the parameter was measured prior to the analysis of flesh firmness using a simple digital imaging method (*Yam & Papadakis, 2004*). Specifically, a fresh peach was placed in a white container inside a lighted box, which was illuminated by two 26 W fluorescent lamps (lumen = 1250 ± 20%). The lamps (13 cm in length) were situated 45 cm above the peach sample. A high-resolution digital camera (Canon XUS105, 12 megapixels, 4 × digital zoom) was used to capture images, which were stored on an Acer T6500 laptop (4.0 GB RAM, 320 GB hard disk). The digital camera was located vertically over the background at a distance of 45 cm. The angle between the camera lens and the lighting source axis was approximately 90°. The camera was fixed on top of the lighting box (Fig. 2), which was constructed from white plastic.

Twenty peach images were captured for each cultivar, and peach color was quantified in the images using the Histogram window in Photoshop (Ado, 2002). The mean color values of lightness (*L*), *a*, and *b* were determined, and these nonstandard color values were converted to *L*^∗^, *a*^∗^, and *b*^∗^, respectively, using the following formulas (*Yam & Papadakis, 2004*):

(1) \begin{eqnarray*}{L}^{\ast }& = \frac{\text{Lightness}}{255} \times 100\end{eqnarray*}

(2) \begin{eqnarray*}{a}^{\ast }& = \frac{240a}{255} -120\end{eqnarray*}

(3) \begin{eqnarray*}{b}^{\ast }& = \frac{240b}{255} -120\end{eqnarray*}

Given that the *L*^∗^, *a*^∗^, and *b*^∗^ coordinate axis defines the three dimensional CIE color space (*Kortei et al., 2015*) in which is the parameters represent lightness, redness, and yellowness, respectively, positive values of *b*^∗^ indicate yellow color; thus, peaches with high *b*^∗^ values are considered high quality.

### Artificial neural network

An ANN can be used as a data processing tool that simulates the learning technique of a biological neural network. ANNs originate from the human nervous system, which comprises a hugely parallel interconnection of nodes that initiate various perceptual and recognition tasks in a small amount of time (*Halagundegowda & Singh, 2018*). Multilayer feed-forward neural networks contain more than one layer of artificial nodes, which allow unidirectional forward connections of inputs and outputs; these networks are also known as multilayer perceptrons (MLPs). MLPs comprise regular input signals, an output neural layer, and a number of hidden layers, with different nodes found between the input and output layers.

**Figure 3:**
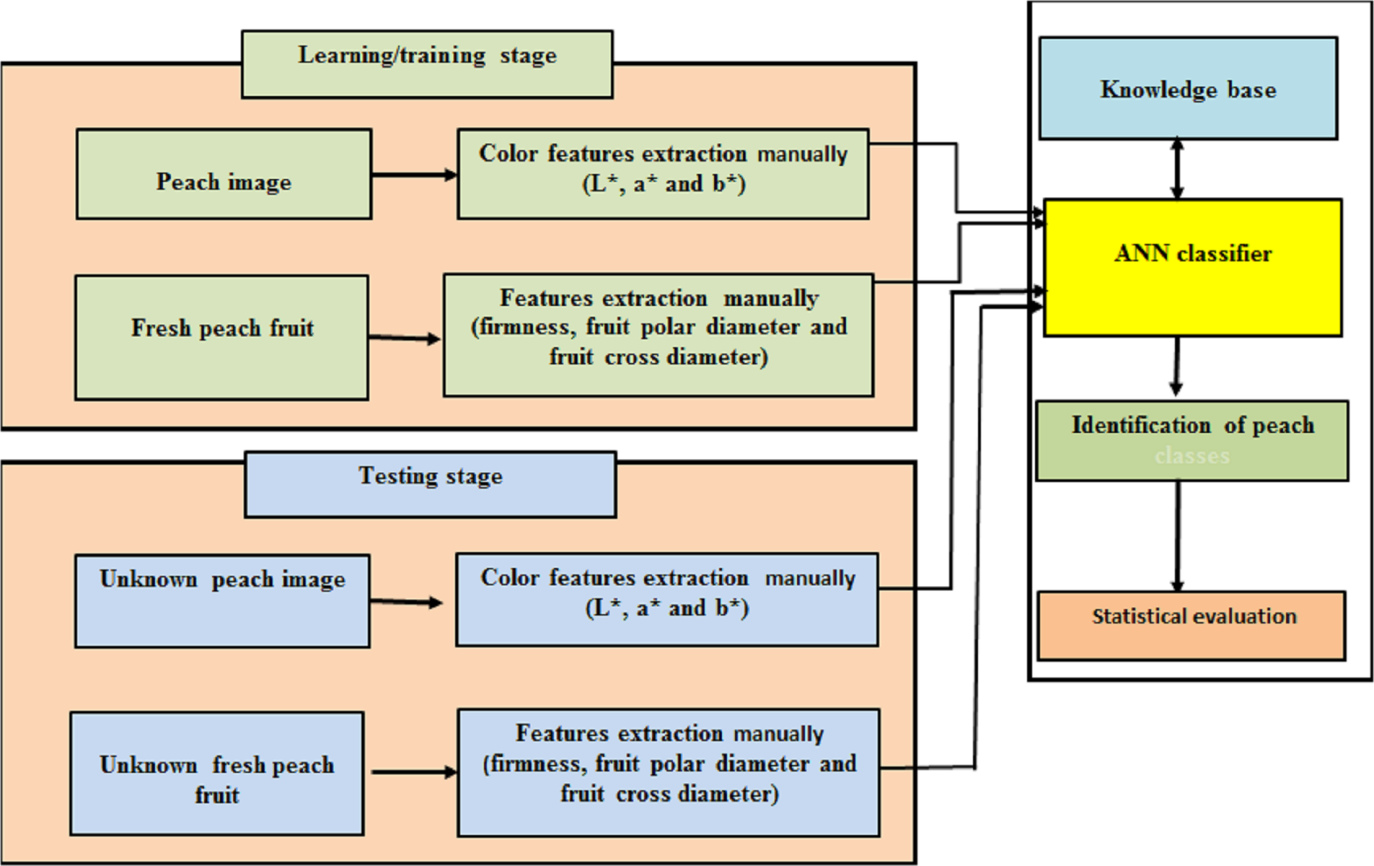
The block diagram of proposed methodology for identification and classification of peach varieties.

In multilayer feed-forward networks, information is transmitted from the input layer to the output layer, as is the case in the human brain where signals move in one direction. Feed-forward networks use any Boolean function and are guaranteed to reach stability provided that the number of hidden nodes is sufficiently large. Indeed, MLPs can be considered as special cases of non-linear regression models. In economics, finance, and agriculture, not all relationships are direct; hidden layers form indirect relationships between input and output variables. Due to the absence of information on the parameters in hidden layers, ANNs are often termed “black boxes”. The number of hidden layers and the number of nodes in each hidden layer relates to the ability of the network to approximate more complex functions. However, networks with complex structures do not necessarily perform better. The block diagram of proposed methodology illustrating the procedure for identification and classification of peach varieties is shown in Fig. 3.

**Figure 4:**
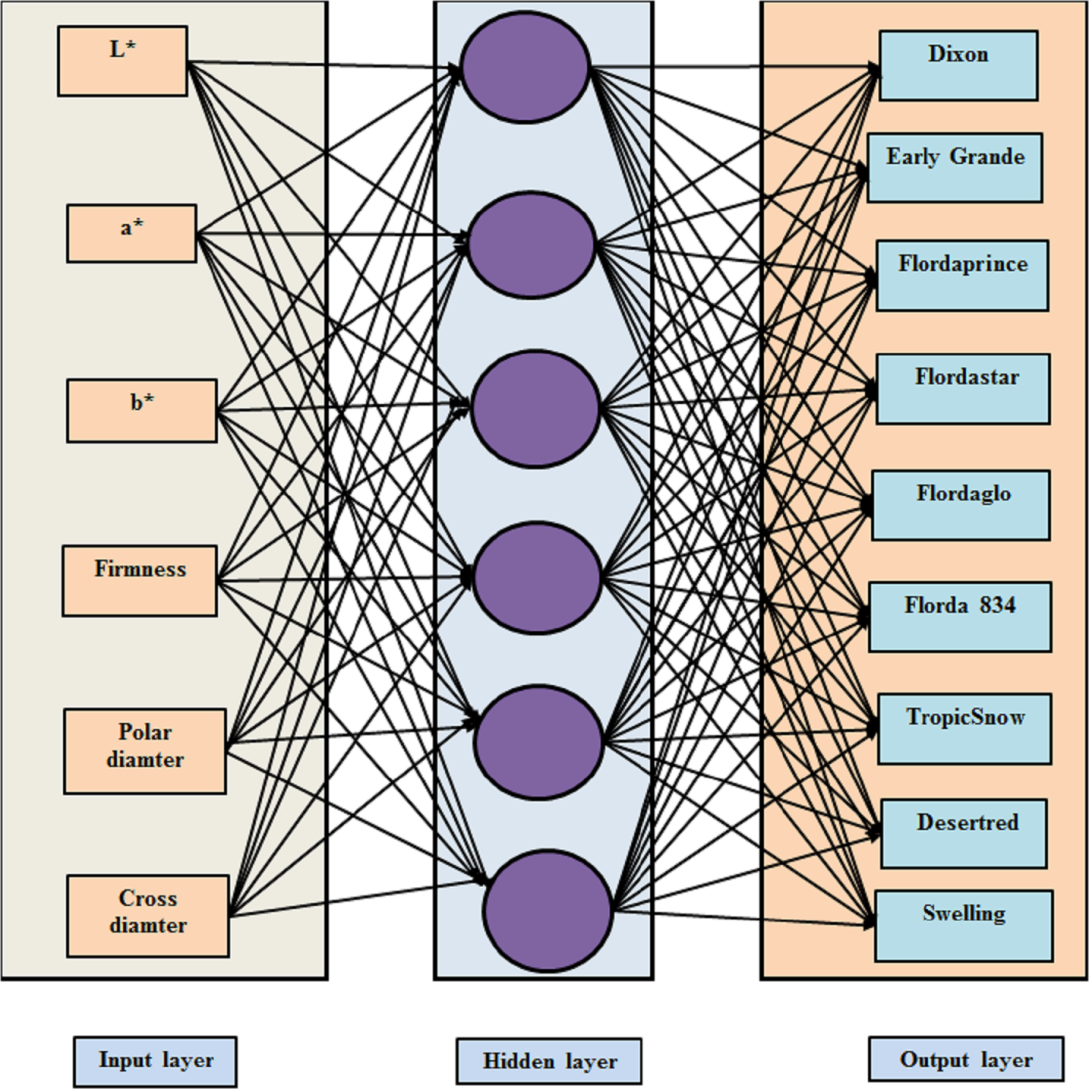
Architectural graph of the multilayer perceptron used in this study.

In the present study, a MLP neural network was fitted to the data using SPSS 19.0. The number of hidden nodes was changed; thus, different neural network model structures were tested before the final architecture of the model was confirmed. Ultimately, a neural network model with six input nodes and five hidden nodes performed better than the other competing models in terms of out-of-sample predictions and identification of peach cultivars (Fig. 4). In the analysis, the peach cultivars were encoded as follows: Class = 1.0 for Dixon, Class = 2.0 for Early Grande, Class = 3.0 for Flordaprince, Class = 4.0 for Flordastar, Class = 5.0 for Flordaglo, Class = 6.0 for Florda 834, Class = 7.0 for TropicSnow, Class = 8.0 for Desertred, and Class = 9.0 for Swelling

### Statistical analysis

Peach cultivar measurements were assessed via ANOVA using SAS software. To compare the mean values of skin color, fruit firmness, and peach diameters, Least Significant Difference was used at significance level of *p* ≤ 0.05.

## Results and Discussion

### Characteristic parameters of peaches

Table 1 lists the measured *L*^∗^, *a*^∗^, and *b*^∗^ values of the nine tested peach varieties. Similar patterns were observed, with *L*^∗^ as the highest value, a mid-range *a*^∗^ value, and a high *b*^∗^ value. The Early Grande and Desertred cultivars had the highest and lowest average *L*^∗^ values at 60.04 ± 1.33 and 36.76 ± 0.16, respectively, with the lower value perhaps caused by browner coloration (*Juncai et al., 2015*). Dixon, Flordastar, Flordaglo, and Swelling cultivars had similar mean *L*^∗^ values (Table 1): 54.33 ± 1.51, 55.78 ± 1.26, 51.72 ± 1.11, and 57.05 ± 0.93, respectively. However, for other peach cultivars, *Montero-Prado, Bentayeb & Nerín (2013)* reported *L*^∗^ values in the range of 71.8 ± 1.9 to 62.4 ± 2.5.

**Table 1:** The measured *L*^∗^, *a*^∗^, and *b*^∗^ of the ninetested peachvarieties.

Sample name	*L* ^∗^			*a* ^∗^			*b* ^∗^		
	Max.	Min.	Mean ± SD	Max.	Min.	Mean ± SD	Max.	Min.	Mean ± SD
Dixon	57.00	51.40	54.33 ± 1.51d	21.15	13.30	17.35 ± 1.64g	51.76	45.51	49.48 ± 1.50a
Early Grande	61.78	57.12	60.04 ± 1.33a	11.75	8.32	9.81 ± 1.05i	41.94	36.85	39.20 ± 1.34c
Flordaprince	49.04	43.92	46.91 ± 1.27g	32.74	27.69	30.91 ± 1.26b	44.07	37.15	40.12 ± 1.66b
Flordastar	58.63	53.67	55.78 ± 1.26c	30.98	24.96	28.52 ± 1.57d	41.12	34.25	38.41 ± 1.51dc
Flordaglo	53.41	49.91	51.72 ± 1.11e	29.81	23.95	27.11 ± 1.54e	40.79	37.86	38.77 ± 0.73dc
Florda 834	45.84	44.47	45.07 ± 0.35h	31.62	27.63	29.95 ± 1.11c	32.61	32.03	32.23 ± 0.15f
TropicSnow	50.62	45.50	47.74 ± 1.43f	25.53	20.47	23.04 ± 1.12f	39.70	33.20	37.04 ± 1.90e
Desertred	37.00	36.44	36.76 ± 0.16i	41.23	37.76	39.71 ± 0.90a	39.63	36.49	38.10 ± 0.85d
Swelling	58.85	55.69	57.05 ± 0.93b	18.30	14.06	16.35 ± 1.07h	33.60	27.79	31.46 ± 1.51f
LSD (0.05)			0.72			0.79			0.86

**Notes.**

SD standard deviation LSD least Significant Difference

Means within a column followed by different letters are significantly different (*P* ≤ 0.05).

In the present study, differences were observed in *a*^∗^ and *b*^∗^ values, where *a*^∗^ represents “-green” to “+red” and *b*^∗^ represents “-blue” to “+yellow”. The Dixon and Swelling cultivars had *a*^∗^ values of 17.35 ± 1.64 and 16.35 ± 1.07, Flordastar and Flordaglo had *a*^∗^ values of 28.52 ± 1.57 and 27.11 ± 1.54, respectively. The Desertred cultivar had the highest average *a*^∗^ value of 39.71 ± 0.90, indicating that it was generally more intensely red than the other cultivars, whereas the Early Grande cultivar had the lowest average *a*^∗^ value of 9.81 ± 1.05. The red coloration of fruit skin is mainly due to the accumulation of anthocyanins (*Lyu et al., 2017*). For *b*^∗^, Flordastar, Flordaglo, and Desertred cultivars had similar average values of 38.41 ± 1.51, 38.77 ± 0.73, and 38.10 ± 0.85, respectively. The average *b*^∗^ of Dixon was higher at 49.48 ± 1.50; as the chlorophyll levels decreased (*Juncai et al., 2015*), however, positive values of *b*^∗^ indicate yellow color; thus, Dixon cultivar which had high *b*^∗^ values is considered high quality. Finally, none of the *a*^∗^ and *b*^∗^ values for the investigated peach cultivars were negative. Our results were in accordance with those reported by Di Vaio et al. (2015). Additionally, *Lyu et al. (2017)* studied the characteristics of 18 cultivars of white-flesh peach fruits cultivated in North China and found that peel *L*^∗^, *a*^∗^, and *b*^∗^ values were 34.01–52.97, 8.36–22.9, and 7.76–15.66, respectively. It is difficult identify peach cultivars individually based on *L*^∗^, *a*^∗^, and *b*^∗^ values because they are too similar to allow classification. In a variation examination of the fruit quality indices of the Xiahui 8 peach variety, *Zhang et al. (2017)* reported mean *L*^∗^, *a*^∗^, and *b*^∗^ values of 69.36 ± 4.78, 15.36 ± 5.71, and 23.31 ± 1.81 when fruit harvesting maturity was degree I and 53.25 ± 4.79, 31.61 ± 3.14, and 18.31 ± 1.6, when it was degree II, respectively. They suggested that the *a*^∗^/*b*^∗^ value represented the true color of the peach, and showed that it was 2.54-fold higher in the degree II fruit relative to the degree I Xiahui 8 peach fruit.

Statistical analysis showed that each color parameter (*L*^∗^, *a*^∗^, and *b*^∗^) of each peach variety was significantly different from that of the other varieties (*p* ≤ 0.05) (Table 1). The range of the coefficient of variation (CV) among the color values was 0.44%–10.70%, indicating that the degree of color indicators varied. The largest CV was observed for *a*^∗^ (10.70%) while the smallest CV was for *L*^∗^ (0.44%). Similarly, *Lyu et al. (2017)* revealed that *a*^∗^ and *L*^∗^ had the highest and lowest CV values in 18 cultivars of white-flesh peaches.

Table 2 presents the measures of firmness, polar diameter, and cross diameter of the nine tested peach varieties. Peach size is a vital quality for consumer preference (*Khezri, Rashidi & Gholami, 2012*), with consumers typically preferring fruits of equal size and shape. The mean cross diameter of samples was 5.54–6.50 cm, whereas the polar diameter was 5.51–6.39 cm (Table 2). *Sherman et al. (1982)* showed that Flordaprince peach fruits are relatively large, averaging over five cm in diameter. However, the same cultivar was smaller when grown under Egyptian climatic conditions, with *Mansour & Stino (1989)* reporting an average diameter of 4.8–4.9 cm and *Shaltout (1987)* reporting an average diameter of 5.2–5.5 cm. Moreover, *Mansour & Stino (1986)* noted that Early Grande peach fruits were medium–large, averaging 5.5–5.9 cm in diameter and 5.5–5.8 cm in length. In addition, *Shama (2000)* showed that the Early Grande peach fruit had a significantly larger diameter and length than other cultivars. Furthermore, *Neelam & Muhammed (2002)* evaluated 15 peach cultivars and showed that Loring peaches had a maximum fruit length of 5.15 cm while Early Grande peaches had the highest fruit diameter of 5.51 cm. Additionally, *Zayan et al. (2015)* showed that for Flordaprince peach, the fruit length and fruit diameter in season 2007 were 5.27 cm and 5.56 cm, respectively, for Early Grande, they were 5.56 cm and 6.01 cm, respectively and for Desertred, they were 5.47 cm and 5.89 cm, respectively.

**Table 2:** The measured firmness, polar diameter, and cross diameter of nine peach varieties.

Sample name	Firmness (Lb/inch^2^)			Polar diameter (cm)			Cross diameter (cm)		
	Max.	Min.	Mean ± SD	Max.	Min.	Mean ± SD	Max.	Min.	Mean ± SD
Dixon	5.32	3.61	4.73 ± 0.43g	5.95	5.88	5.92 ± 0.02d	5.57	5.53	5.54 ± 0.01f
Early Grande	7.10	6.18	6.69 ± 0.24e	6.32	5.95	6.15 ± 0.10b	5.83	5.76	5.79 ± 0.02e
Flordaprince	7.65	6.81	7.29 ± 0.26d	5.78	5.62	5.72 ± 0.04e	6.19	5.52	5.85 ± 0.19ed
Flordastar	6.58	6.07	6.28 ± 0.14f	5.72	5.41	5.51 ± 0.08f	5.94	5.68	5.79 ± 0.07e
Flordaglo	6.65	6.24	6.48 ± 0.10fe	6.19	6.03	6.12 ± 0.04b	5.97	5.78	5.87 ± 0.06d
Florda 834	8.74	7.73	8.48 ± 0.32c	6.05	5.98	6.02 ± 0.02c	6.11	5.85	5.93 ± 0.07c
TropicSnow	12.27	12.03	12.16 ± 0.07a	6.47	6.21	6.39 ± 0.06a	6.49	6.02	6.31 ± 0.13b
Desertred	13.22	9.91	11.48 ± 0.83b	6.97	5.96	6.43 ± 0.25a	6.76	6.24	6.50 ± 0.13a
Swelling	12.98	11.23	12.07 ± 0.48a	5.86	5.64	5.75 ± 0.06e	5.98	5.74	5.85 ± 0.06ed
LSD (0.05)			0.24			0.06			0.06

**Notes.**

SD standard deviation LSD least Significant Difference

Means within a column followed by different letters are significantly different (*P* ≤ 0.05).

The firmness of fruit flesh is also an important factor of fruit quality (*Maness, Brusewitz & McCollum, 1992*). Firmness in peaches can be an indicator of immaturity or over maturity. For example, excessive firmness indicates an immature peach in which the mesocarp is tightly bound to the stone with little free juice (*Armstrong, Stone & Brusewitz, 1997*). Fruit firmness is considered low when the value is between 0.40 and 0.62 kg/cm^2^ (Santiago-Mejía et al, 2008). In the present study, fruit flesh firmness ranged from 4.73 to 12.16 Lb/inch^2^ (i.e., 1.65–4.24 kg/cm^2^) at the time of commercial harvest (Table 2). Maximum levels of fruit firmness for marketing fresh peaches and nectarines are set by the EU at a 6.5 kg (= 63.7 N)/0.5 cm^2^ (eight mm diameter probe) (Commission Regulation (EC) No. 1861/2004 of October 28, 2004). Significant differences in firmness were observed among some of the nine tested peach cultivars (*p* ≤ 0.05). TropicSnow peaches had the highest firmness values at 12.16 Lb/inch^2^, which was significantly higher than that of other peach varieties. Although there were significant differences in firmness among the cultivars, there was no obvious trend among the nine peach varieties (Table 2), even though the picking period for all cultivars was around mid-May to the end of May. There was less difference in firmness among the cultivars Dixon, Early Grande, Flordaprince, Flordastar, Flordaglo, and Florda 834 compared with the cultivars TropicSnow, Desertred, and Swelling; this was likely due to the cultivars in these groups having the same pattern of ripening despite their differences, indicating that firmness is highly cultivar-dependent. *Zayan et al. (2015)* showed that for Flordaprince, Early Grande, and Desertred peaches, the firmness were 12.79, 14.86, and 12.96 Lb/inch^2^, respectively in season 2007. In addition, *Ahmed, El-Habashy & Maklad (2017)* reported the firmness for Flordaprince and Swelling peaches budded on Nemaguard rootstock were 8.914 and 5.82 Lb/inch^2^, respectively in season 2014. However, changes in fruit tissue firmness during ripening have been largely attributed to enzymic dissociation of the cell walls between adjacent cells (*Huber, 1983; Fischer & Bennett, 1991).* Soft peaches can be excessively juicy (*Armstrong, Stone & Brusewitz, 1997*), while a decline in peach fruit firmness is observed as background color becomes more yellow and less green (*Bonora, Stefanelli & Costa, 2013*).

**Figure 5:**
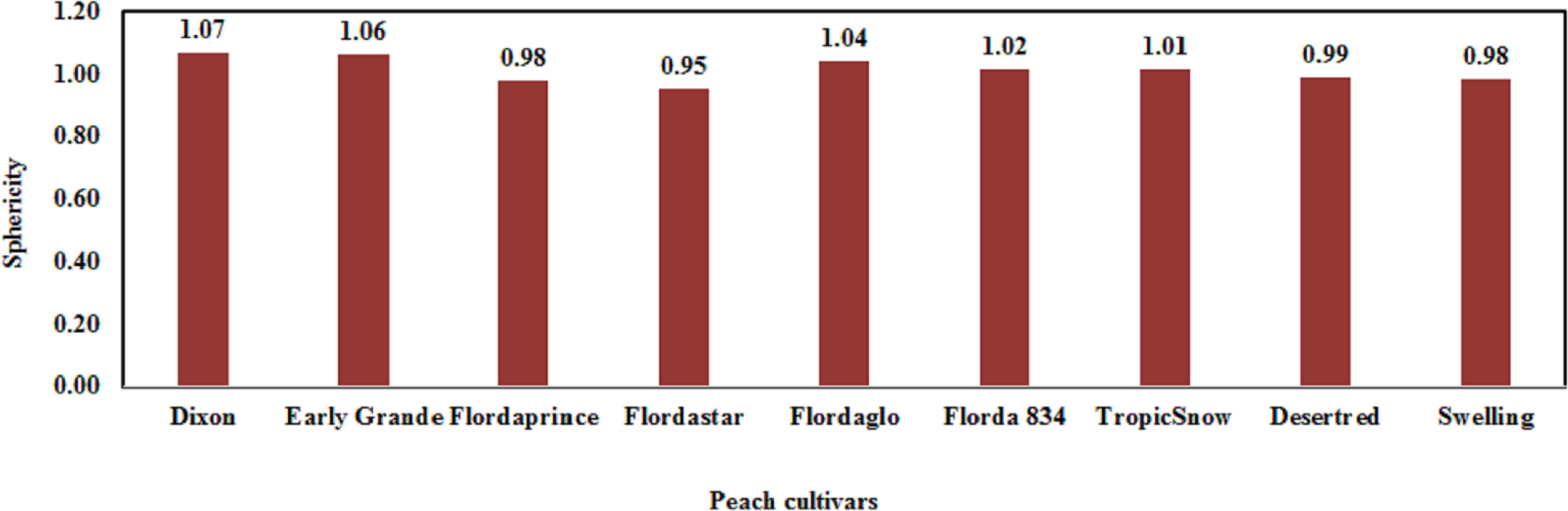
The fruit sphericity of the nine tested peach cultivars.

The sphericity of the peaches (polar diameter/cross diameter) was 0.95–1.07 (Fig. 5). Sphericity values <0.9 indicate that the fruit is oblate, whereas values >1.1 indicate that it is oblong; the remaining fruits with intermediate index values are considered round (*Buyanov & Voronyuk, 1985*). As shown in Fig. 5, the Dixon and Early Grande cultivars were oblong whereas the Flordaprince, Flordastar, Desertred, Swelling, Flordaglo, Florda 834, and TropicSnow cultivars were round. Thus there was some dissimilarity in the shape of the studied cultivars. In particular, Dixon was the least spherically shaped variety. *Zayan et al. (2015)* showed that for Flordaprince, Early Grande, and Desertred peaches, the shape rate which is a function of fruit length and diameter of the fruit in season 2007 was 0.95, 0.93, and 0.93, respectively.

Statistical analysis of firmness, polar diameter, and cross diameter showed that each parameter of each variety was significantly different from the parameters of other varieties (*p* ≤ 0.05) (Table 2). According to Commission Regulation (EC) no. 1861/2004, all studied peach cultivars met the minimum diameter to be considered in the “extra” category (i.e., 56 mm).

### Performance of the ANN classifier

As shown in Table 3, 112 cases were assigned to the training dataset and 68 patterns were used as the testing dataset (conforming to rules that 62.2% of data should be used for training while 37.8% of data is used for testing). The training dataset comprised data used to train the neural network; the testing dataset was an independent set of data used to track errors during training and prevent overtraining.

**Table 3:** Case processing summary.

		N (No. of patterns)	Percentage
Sample	Training	112	62.2%
	Testing	68	37.8%
Valid		180	100.0%
Excluded		0	
Total		180	

Table 4 provides details of the neural network’s architecture. Six input variables were used as covariates in analysis and the standardized rescaling method was used to adjust the covariates. Scale-dependent variables and covariates were rescaled by default to improve network training. All rescaling was performed based on the training data, even if a testing dataset was defined. The network had an input layer with six input nodes, with the number of nodes in the input layer being the number of covariates. A single hidden layer had six hidden nodes and an output layer had one output with nine nodes. A hyperbolic tangent activation function was used in the hidden layers, taking real valued arguments and then transforming them into a range (−1 to 1). The error was the cross entropy error because the softmax activation function was applied to the output layer. It took a vector of real valued arguments and transformed it to a vector for which the elements fell within a range (0 to 1) and summed to 1. Softmax can be used only if all dependent variables are categorical. Training time was very small (00:00:00.054 s), however, *Livni, Shalev-Shwartz & Shamir (2014)* pointed out that training time is defined as how much computation time is required to learn the class. On the same line, other research papers presented training time of 00:00:00.001s (Pandurangan et al., 2017), 0:00:00.000 s (*Maliki et al., 2011*), and 0:00:00.265 s (*Alabi, Issa & Afolayan, 2013*). Additionally, when training a neural net with one hidden layer of size five and one input node for 100 steps, a variety of training times ranging from around 0.808 to 1.04 s could be recorded (*Adlers & Pihl, 2018*). Generally, the training time depends completely on the structure of used ANN.

**Table 4:** Network information summary.

Input layer	Covariates	1	*L* ^∗^
		2	*a* ^∗^
		3	*b* ^∗^
		4	Firmness
		5	Polar diameter
		6	Cross diameter
	Number of units^a^	6
	Rescaling method for covariates		Standardized
Hidden layer(s)	Number of hidden layers		1
	Number of units in hidden layer 1^a^		6
	Activation function		Hyperbolic tangent
Output layer	Dependent variables	1	Class
	Number of units		9
	Activation function		Softmax
	Error function		Cross entropy

**Notes.**

a Excluding the bias unit.

Table 5 shows the results of training and applying the final ANN model to the testing dataset. The cross entropy error is presented because the output layer uses the softmax activation function; the ANN attempts to minimize this error function during the training process. The cross entropy error has a predicted value for each category, where each predicted value is the probability that the case belongs to the category. As shown in Table 5, the cross entropy error was 0.165, which is an allowable level that enables analysis to continue into further steps. The percentage of incorrect classifications can be taken from the classification table, in which 0% of classifications were mismatched with the original observed samples. The estimation algorithm ended because the training error ratio criterion (0.001) was achieved (Table 5). Ideally, training would end when the error had converged. The cross entropy error was 0.140 with 0% incorrect classifications of the testing data.

**Table 5:** Model summary.

Training	Cross entropy error	0.165
	Percentage of incorrect predictions	0.0%
	Stopping rule used	Training error ratio criterion (0.001) achieved
	Training time	00:00:00.054 s
Testing	Cross entropy error	0.140
	Percentage of incorrect predictions	0.0%

To the best of our knowledge, almost all research results related to the identification of peach fruit have taken offline images and none of the studies performs in real-world scenario with acceptable accuracy. However, in the present study, the overall classification accuracy was 100% as shown in Table 6 in the training and testing phases and this mean that using the selected features, the peach varieties could be recognized easily. No overfitting is occurred, however, overfitting is a major problem in supervised machine learning which avoids us from perfectly generalizing the models to well fit experimental data on training dataset, in addition to hidden data on testing dataset (*Ying, 2019*). As a consequence of the existence of noise, the complexity of classifiers, and the limited size of training set, overfitting occurs. To decrease the effects of overfitting, several approaches are suggested to address to these causes as reported by *Ying (2019).*

**Table 6:** Confusion matrix for peach varieties using training and testing datasets.

	Observed	Predicted									
		Dixon	Early Grande	Flordaprince	Flordastar	Flordaglo	Florda 834	TropicSnow	Desertred	Swelling	Percentage correct
Training	Dixon	12	0	0	0	0	0	0	0	0	100%
	Early Grande	0	14	0	0	0	0	0	0	0	100%
	Flordaprince	0	0	13	0	0	0	0	0	0	100%
	Flordastar	0	0	0	12	0	0	0	0	0	100%
	Flordaglo	0	0	0	0	14	0	0	0	0	100%
	Florda 834	0	0	0	0	0	12	0	0	0	100%
	TropicSnow	0	0	0	0	0	0	14	0	0	100%
	Desertred	0	0	0	0	0	0	0	13	0	100%
	Swelling	0	0	0	0	0	0	0	0	8	100%
	Overall Percentage	10.7%	12.5%	11.6%	10.7%	12.5%	10.7%	12.5%	11.6%	7.1%	100%
Testing	Dixon	8	0	0	0	0	0	0	0	0	100%
	Early Grande	0	6	0	0	0	0	0	0	0	100%
	Flordaprince	0	0	7	0	0	0	0	0	0	100%
	Flordastar	0	0	0	8	0	0	0	0	0	100%
	Flordaglo	0	0	0	0	6	0	0	0	0	100%
	Florda 834	0	0	0	0	0	8	0	0	0	100%
	TropicSnow	0	0	0	0	0	0	6	0	0	100%
	Desertred	0	0	0	0	0	0	0	7	0	100%
	Swelling	0	0	0	0	0	0	0	0	12	100%
	Overall Percentage	11.8%	8.8%	10.3%	11.8%	8.8%	11.8%	8.8%	10.3%	17.6%	100%

Regardless of the ANN models are performing better in its application to human problems, there is an increasing need to address the problem of adopting a regular methodology in ANNs development stage to increase its performance (*Abiodun et al., 2018*). For instance, a potential way to improve the robustness of the ANN is to increase the volume of training data and use both good and bad quality data as training samples. However, model robustness means predictive capability of ANN types in generalizing range of data like those used for training (*Abiodun et al., 2018).* On the other hand, the image grading system is simple and efficient and can be considered a suitable first stage to mechanizing the commercial grading of fruits (*Momin et al., 2017*). Thus, in fruit classification automation systems, which are based on color features extracted from fruit images, the noise in images can be eliminated using *image* processing *algorithm* (*Momin et al., 2017*) to control a process at optimum efficiency (*Patel et al., 2012*). Moreover, the number of training cases is one of various factors which can affect the optimum performance of an ANN classifier (Sildir, Aydin & Kavzoglu, 2020). The vital idea for dealing with small datasets is to maximize the extension of the training set by a specific facility of the tool, the so-called all-frame or leave-one-out cross validation procedure (*Pasini, 2015*).

The ANN classifier could be a very useful tool in identification of peach varieties based on skin color, size and firmness. Thus, it will enable to manufacture more easily, simply and economically an equipment in real-world to be developed thanks to this model. It would be possible to construct automation systems, using the ANN classifier developed, for identification peach varieties to use in supermarkets or grading stations.

### Sensitivity analysis

**Figure 6:**
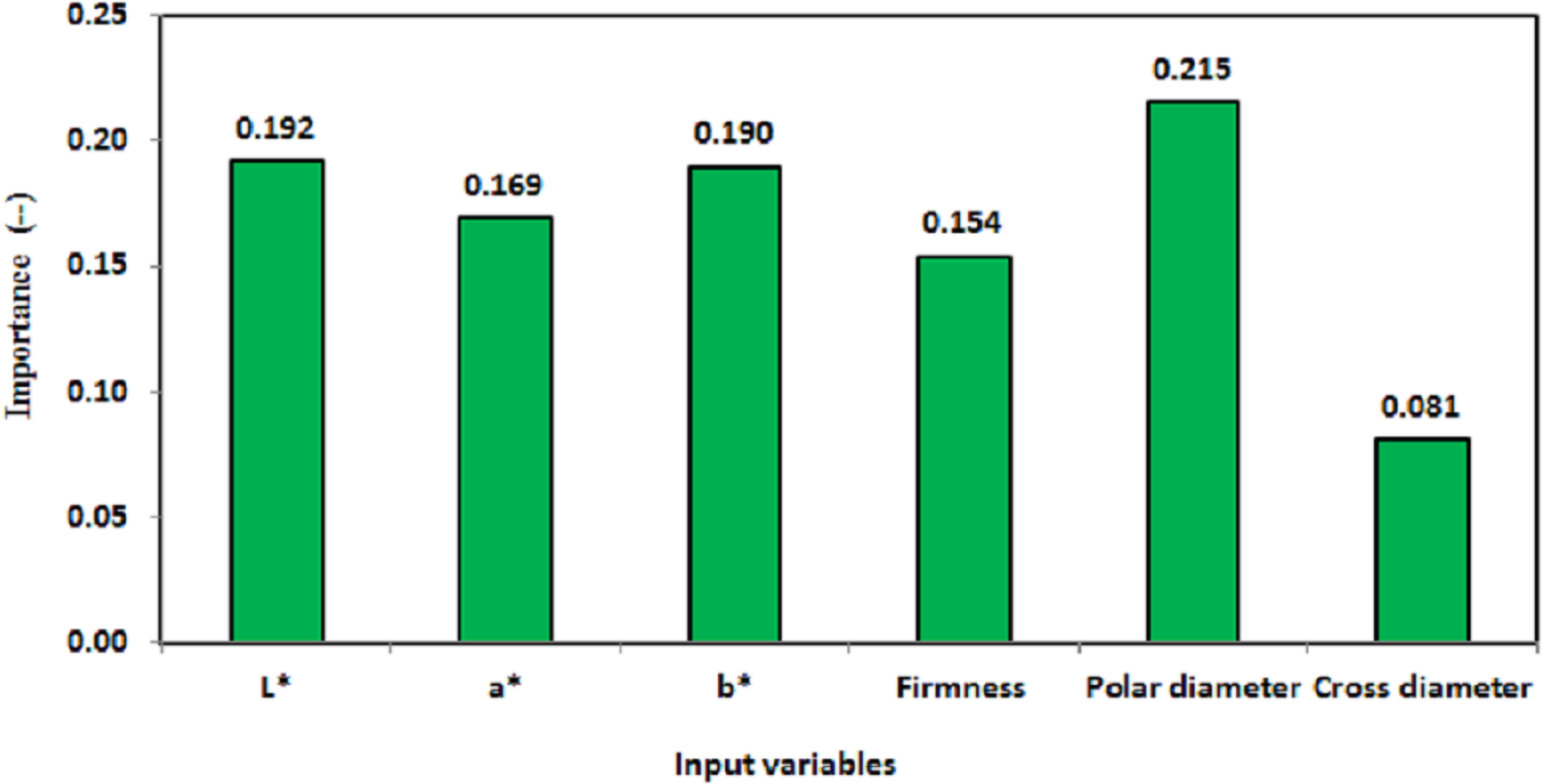
Importance of the peach characteristic variables to the identification of cultivars.

**Figure 7:**
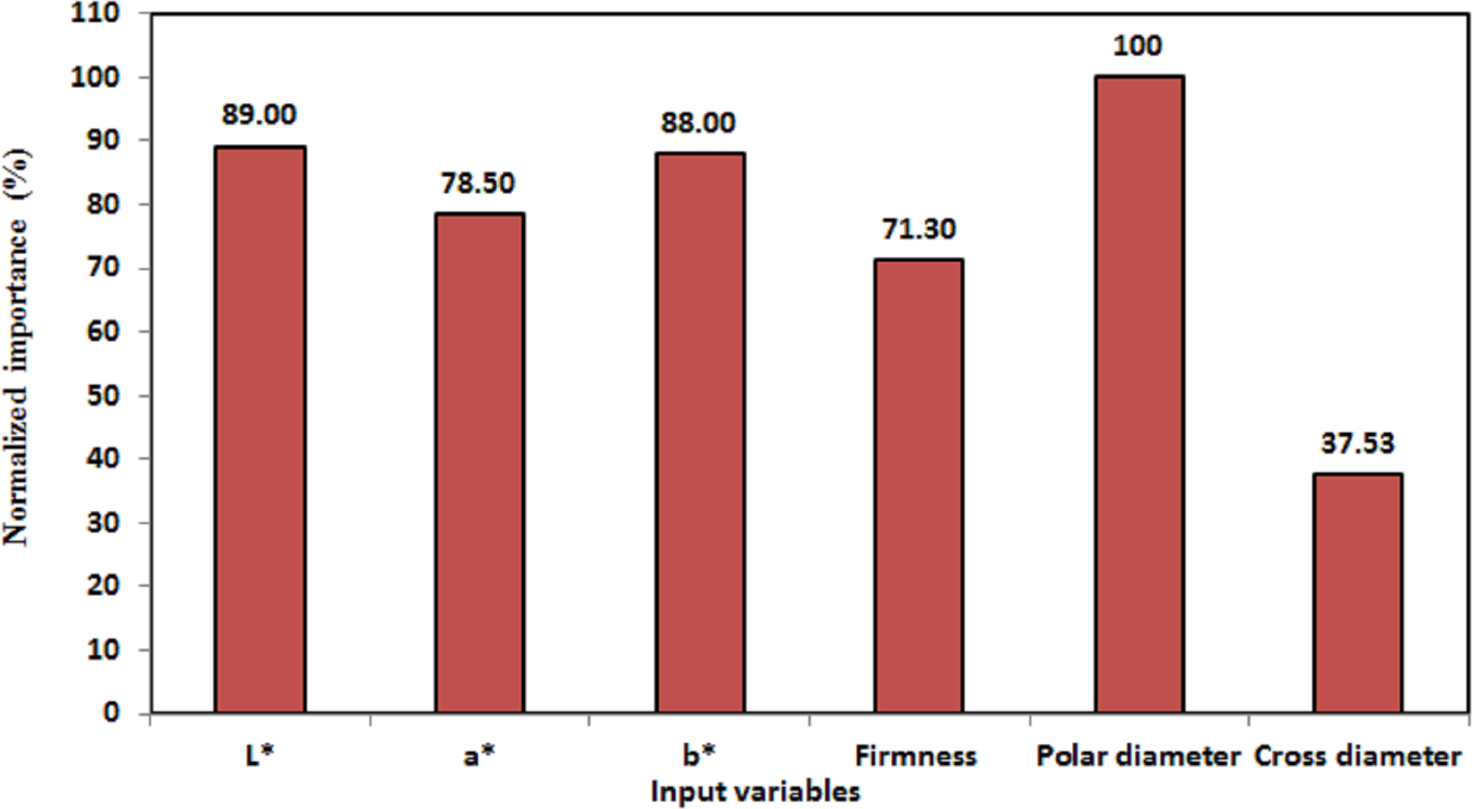
Normalized importance of the peach characteristic variables to the identification of cultivars.

Sensitivity analysis (*Kakhki, Freeman & Mosher, 2019*) can be used to appraise the relationship between the input attributes and outcomes as a feature of the extraction method in ANN models (*Harrington & Wan, 2002*). Such analysis calculates the significance of each attribute (independent variables) in determining the probability of an output in the neural network (IBM, Released 2010). Figure 6 presents the importance of all attributes used in the present study. However, the importance of an independent variable is a measure of how much the network’s model-predicted value changes for different values of the independent variable (*Alabi, Issa & Afolayan, 2013*). Additionally, Fig. 7 presents the normalized importance of all attributes used in the present study. By inspection of Fig. 6,the order of the importance of the input variables that had the greatest effect on identification of peach cultivars was as follows: polar diameter (0.215), *L*^∗^ (0.192), *b*^∗^ (0.190), *a*^∗^ (0.169), firmness (0.154), and cross diameter (0.081).

Also, by inspection of Fig. 7, the order of the normalized importance of the input variables that had the greatest effect on identification of peach cultivars was as follows: polar diameter (100%), *L*^∗^ (89.0%), *b*^∗^ (88.00%), *a*^∗^ (78.50%), firmness (71.30%), and cross diameter (37.53%). Thus, polar diameter was significantly important for the potential identification of peaches, which is in agreement with the findings of a previous study by *Naskar & Bhattacharya (2015)* on the significance of work-related activities in fruit classification, in which geometric and color attributes were used; these authors showed that the color and shape, shape and texture, or texture and color attributes were acceptable variables for fruit recognition. Therefore, as the number of investigated variables increases, there is a greater possibility of identification and the model becomes easier to interpret. The findings of this study have to be seen in light of some limitations. The peach identification accuracy is based on measurements of polar diameter, *L*^∗^, *b*^∗^, and *a*^∗^ of peach skin, firmness, and cross diameter for definite cultivars. These measurements, therefore, subjected to biases and confounding that may have influenced our identification accuracy.

## Conclusions

In this study, the color values of skin, polar and cross diameters, and firmness of nine peach varieties were evaluated for the identification of these cultivars using an ANN classifier. Each parameter of each variety was significantly different from the parameters of other varieties. Moreover, all studied peach cultivars met the minimum diameter for the fruit to be considered in the “extra” category. We conclude that the ANN algorithm is useful for supervised peach identification based on values of color, polar and cross length, and firmness. Strong results were achieved in the training and testing phases; these generally showed an intrinsic distinction between peach cultivars. Indeed, the classification of peach cultivars based on the ANN had an accuracy of 100% using a hyperbolic tangent function. We have developed and tested ANN but other data mining techniques can participate in this research field in particular to have a programmed machine for identification peach varieties in real time, which had not previously been proposed in the literature.

##  Supplemental Information

10.7717/peerj.11529/supp-1 Supplemental Information 1 Field study raw measurements

## Additional Information and Declarations

### Data Availability

The following information was supplied regarding data availability: The raw measurements are available in the Supplementary File.
